# Neuroprotection against Aminochrome Neurotoxicity: Glutathione Transferase M2-2 and DT-Diaphorase

**DOI:** 10.3390/antiox11020296

**Published:** 2022-01-31

**Authors:** Juan Segura-Aguilar, Patricia Muñoz, Jose Inzunza, Mukesh Varshney, Ivan Nalvarte, Bengt Mannervik

**Affiliations:** 1 Molecular and Clinical Pharmacology ICBM, Faculty of Medicine, University of Chile, Santiago 8380000, Chile; 2 Instituto de Ciencias Biomédicas, Facultad de Ciencias de la Salud, Universidad Autónoma de Chile, Santiago 8900000, Chile; pams@gmail.com; 3 Department of Biosciences and Nutrition, Karolinska Institutet, SE-14157 Huddinge, Sweden; jose.inzunza@ki.se (J.I.); mukesh.varshney@ki.se (M.V.); ivan.nalvarte@ki.se (I.N.); 4 Department of Biochemistry and Biophysics, Arrhenius Laboratories, Stockholm University, SE-10691 Stockholm, Sweden; bengt.mannervik@dbb.su.se

**Keywords:** glutathione, glutathione transferase, dopamine, Parkinson’s disease, neuron, astrocytes, neuroprotection, aminochrome

## Abstract

Glutathione is an important antioxidant that plays a crucial role in the cellular protection against oxidative stress and detoxification of electrophilic mutagens, and carcinogens. Glutathione transferases are enzymes catalyzing glutathione-dependent reactions that lead to inactivation and conjugation of toxic compounds, processes followed by subsequent excretion of the detoxified products. Degeneration and loss of neuromelanin-containing dopaminergic neurons in the nigrostriatal neurons generally involves oxidative stress, neuroinflammation, alpha-synuclein aggregation to neurotoxic oligomers, mitochondrial dysfunction, protein degradation dysfunction, and endoplasmic reticulum stress. However, it is still unclear what triggers these neurodegenerative processes. It has been reported that aminochrome may elicit all of these mechanisms and, interestingly, aminochrome is formed inside neuromelanin-containing dopaminergic neurons during neuromelanin synthesis. Aminochrome is a neurotoxic ortho-quinone formed in neuromelanin synthesis. However, it seems paradoxical that the neurotoxin aminochrome is generated during neuromelanin synthesis, even though healthy seniors have these neurons intact when they die. The explanation of this paradox is the existence of protective tools against aminochrome neurotoxicity composed of the enzymes DT-diaphorase, expressed in these neurons, and glutathione transferase M2-2, expressed in astrocytes. Recently, it has been reported that dopaminergic neurons can be protected by glutathione transferase M2-2 from astrocytes, which secrete exosomes containing the protective enzyme.

## 1. Glutathione as Antioxidant

Glutathione is an abundant tripeptide in essentially all aerobic organisms, and a pivotal component in the cellular protection against oxidative stress, electrophilic mutagens, and carcinogens [1]. The thiol group of the γ-l-glutamyl-l-cysteinyl-glycine (glutathione, GSH) can serve as a reductant, scavenge unpaired electrons, and conjugate various toxins and thereby enable their elimination. The biochemistry of glutathione has been extensively covered in a two-volume treatise [2] including a general review of its mechanism of action [3], and a recent review details recent advances with particular emphasis on redox homeostasis in the brain [4]. Glutathione is the most abundant low-molecular-mass thiols in the cell and is present in millimolar tissue concentrations, which reach 10 mM in liver [5]. Glutathione occurs predominantly in the reduced form (normally > 98%) owing to the activity of the ubiquitous NADPH-dependent glutathione reductase [6]. The enzyme shows specificity for glutathione disulfide (GSSG), even though modest activity is obtained with the mixed glutathione-coenzyme A disulfide [7]. In general, disulfides (RSSR) and thiosulfate esters, such as s-sulfoglutathione, are reduced via thiol-disulfide interchange with glutathione (GSH), catalyzed by thioltransferase (also called glutaredoxin 1) [8].

RSSR + 2 GSH → 2 RSH + GSSG

Thus, by coupling to glutathione reductase, efficient reduction of cystine and other disulfides formed by oxidative processes is afforded by glutathione and thioltransferase [9]. Similarly, disulfides in proteins formed under oxidative conditions can be reduced by the same device, as well as by thioredoxin coupled to thioredoxin reductase [10].

Primary products of oxygen metabolism, which include reactive oxygen species (ROS) such as singlet oxygen, superoxide, hydroxyl radical, and hydrogen peroxide are also detoxified by glutathione. Furthermore, a diverse range of toxic secondary products derived from reactions of ROS with cell constituents are inactivated by reactions catalyzed by glutathione transferases and selenium-dependent glutathione peroxidases [11,12]. The harmful products include alkenals, epoxides, quinones, and organic peroxides, and it has been suggested that their toxicity has contributed to the evolutionary trajectories of the glutathione-dependent enzymes.

The biosynthesis of glutathione from its constituent amino acids, glutamic acid, cysteine, and glycine, proceeds via γ-glutamylcysteine catalyzed by γ-glutamylcysteine ligase followed by addition of the C-terminal glycine catalyzed by glutathione synthetase [1]. The first reaction can be rate-limited by either the availability of cysteine or by the activity of the γ-glutamylcysteine ligase, which is negatively feedback regulated by glutathione. Liver is a major site of glutathione biosynthesis, and micromolar concentrations of the thiol circulate in blood plasma, but the blood–brain barrier prevents uptake into the brain. Thus, the precursors have to be taken up by membrane-bound transport proteins for glutathione biosynthesis in the brain tissues. Glutathione plays a pivotal protective role in the nervous system since the brain is subject to oxidative stress and ROS are produced in mitochondria. In addition to the ROS listed above, nitric oxide and superoxide can combine to form peroxynitrite, which can reach farther than other ROS and cause more widespread tissue damage. Both the primary and the secondary products of oxidative stress can be inactivated by glutathione-dependent reactions, and thereby counteract degenerative processes in the brain.

A role of glutathione in the cellular protection against Parkinson’s disease was suggested several decades ago based on the deficiency in substantia nigra [13], but it remains unclear whether disruption of the proper glutathione status is a causative effect or a result of the disease [13]. A decrease in the cellular glutathione concentration in the brain of Parkinson’s disease patients, in particular in the substantia nigra, is well established. On this basis clinical trials involving intravenous [14] or intranasal [15] glutathione administration to patients have been made in order to supplement the endogenous glutathione.

Precursors of glutathione biosynthesis, such as *N*-acetyl-l-cysteine, which have been demonstrated to increase glutathione concentrations following intravenous administration, have also been considered for clinical applications [8,16]. More recently a combination of *N*-acetylcysteine and a regenerative secretome preparation of mesenchymal stem cells has been suggested [17].

The transcription factor nuclear factor erythroid 2-related factor 2 (Nrf2) regulates the coordinated biosynthesis of glutathione and various proteins enabling antioxidant and protective functions. In particular, γ-glutamylcysteine ligase catalyzing the rate-limiting step in glutathione synthesis, several glutathione transferases (GSTs), as well as DT-diaphorase are upregulated by activators of Nrf2 [18]. Experiments suggests that triggering the higher antioxidant potential in astrocytes causes release of glutathione, thereby supporting the less capable neurons in mixed populations [6,19].

Furthermore, the excitatory amino acid carrier 1, the glutamate/cysteine transporter selectively present in neurons, plays a central role in the regulation of neuronal GSH production, and it has been suggested as a new target, in addition to Nrf2, in therapeutic strategies for neurodegenerative diseases [4,20].

## 2. Glutathione Transferase

GSTs are enzymes catalyzing glutathione-dependent reactions that lead to inactivation and conjugation of toxic compounds, processes followed by subsequent excretion of the detoxified products. GSTs occur abundantly in multiple forms and the “GSTome” [21] encompasses both soluble and membrane-bound proteins. The homologous soluble GSTs, which occur in several classes [22], are prominent components of the cellular defense against toxicants. Discovered as enzymes detoxifying xenobiotics, we later found that the natural substrates are primarily products of lipid peroxidation and other noxious compounds derived from endogenous cellular components [11,23,24]. Recent findings relevant to Parkinson’s disease show that α-synuclein oligomers formed with the dopamine-derived aminochrome-glutathione conjugate are not neurotoxic [25]. We have also demonstrated that GSTs can be secreted from cells in culture and taken up in catalytically functional form by other cells [26], suggesting extracellular trafficking of GSTs in tissues.

**Figure 1:**
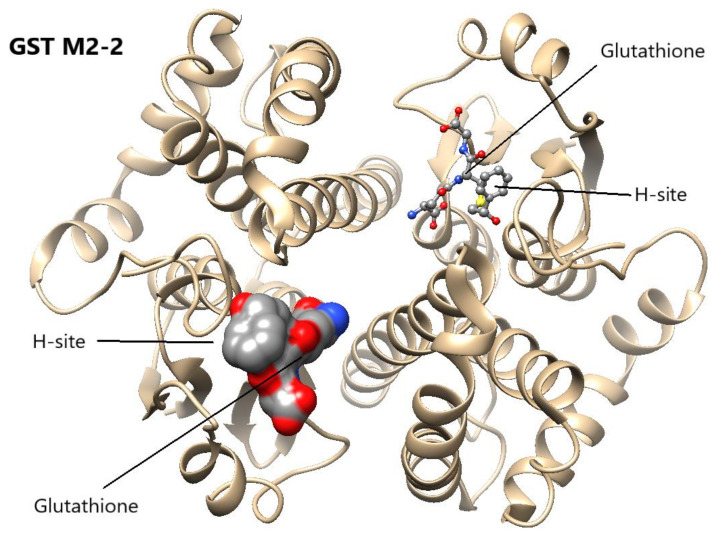
Human GST M2-2 showing location of the G-site and the H-site in each subunit. A structure of the enzyme in complex with aminochrome or its glutathione conjugate is not available, but the location of glutathione (G-site) and the H-site can be identified by the bound glutathione conjugate of S-styrene 7,8-oxide (shown left in space-filling and right in ball-and-stick representations). The figure is based on the crystal structure (PDB ID: 2C4J) and rendered in UCSF Chimera [29].

Crystal structures of the soluble GSTs [27] show that the enzymes are composed of two protein subunits, each carrying a binding site for glutathione (the G-site), as well as a binding site for the electrophilic second substrate (the H-site). The subunits are identical, or in some cases nonidentical but homologous, subunits from the same GST class [28]. The mu class encompasses a cluster of five homologous genes *GSTM1-GSTM5* on human chromosome 1, where *GSTM2,* assigned to cytogenetic band 1p13.3, encodes the homodimeric GST M2-2(Figure 1).

The enzyme GST M2-2 was first purified from human skeletal muscle under the name GST-4 [30] and was later also found at high levels in brain, testis, and heart, but not detectable in liver [31]. The catalytic activity of GST M2-2 with the toxic ortho-quinones derived from catecholamines is several orders higher than those of all other human GSTs suggesting a designated protective role of GST M2-2 against oxidative stress caused by redox cycling of the orthoquinone substrates [32]. The highest specific activity has been observed with aminochrome, but dopachrome and noradrenochrome are also highly active substrates [24]. Notably, GST M2-2 is inducible by treatment with aminochrome, as demonstrated in astrocytoma U373MG cells, and GST M2-2 released from the cells protect dopaminergic neurons from aminochrome [26].

## 3. Parkinson’s Disease Neurodegeneration

Parkinson’s disease is the second most prevalent neurodegenerative disease, which affects neuromelanin-containing dopaminergic neurons, generating motor symptoms such as rest tremors, bradykinesia, rigidity, and postural instability [22]. However, the existence of non-motor symptoms such as hyposmia, depression, sleep disorders, constipation, anxiety, cognitive decline, orthostatic hypotension, and visual disturbances appear several years before the appearance of motor symptoms [33,34,35,36]. The motor symptoms appear when 60–70% of neuromelanin-containing dopaminergic neurons have been lost. The degenerative process and the progression of the disease is extremely slow and takes years to progress from non-motor to motor symptoms, suggesting that environmental or exogenous neurotoxins are not involved in the degenerative process of idiopathic Parkinson’s disease. This idea is supported by the contrasting fact that 1-methyl-4-phenyl-1,2,3,6-tetrahydropyridine (MPTP) developed severe Parkinsonism in just 3 days in drug addicts who used synthetic illegal drugs contaminated with this neurotoxin [37]. In addition, Parkinsonism has been observed in young workers occupationally exposed to manganese in mining or welding [38], copper mining [39], and paraquat in agriculture [40].

Therefore, these findings suggest that in the idiopathic form of the disease, the degenerative process of neuromelanin-containing dopaminergic neurons is not induced by exogenous neurotoxins or prion-like propagative process, as evidenced by the extremely slow degeneration over multiple years [35,40,41]. It seems plausible that the extreme slowness of the degenerative process in idiopathic Parkinson’s disease depends on degeneration focused on a single neuromelanin-containing dopaminergic neuron that ends with its death. This degenerative event is focalized and does not trigger the degeneration of neighboring neuromelanin-containing dopaminergic neurons. At another time, a new and independent neurotoxic event occurs that ends with the loss of a new neuromelanin-containing dopaminergic neuron. These neurotoxic events accumulate over time and after years of the focalized degenerative process motor symptoms finally appear when at least 60–70% of the neuromelanin-containing dopaminergic neurons have been lost. According to this model of neurodegeneration focused on a single neuron, the degenerative process must be triggered by an endogenous neurotoxin that is generated within the neuromelanin-containing dopaminergic neurons.

The identity of the neurotoxin that triggers the degenerative process of the neuromelanin-containing dopaminergic neurons of the nigrostriatal system is unclear. However, there is general agreement in the scientific community that this degenerative process involves oxidative stress, mitochondrial dysfunction, endoplasmic reticulum stress, neuroinflammation, dysfunction of protein degradation of the lysosomal and proteasomal systems, and aggregation of alpha-synuclein to neurotoxic oligomers [33,42,43,44,45,46,47,48]. However, what triggers these neurotoxic mechanisms in idiopathic Parkinson’s disease is still unknown.

The discovery that mutations in some genes that code for proteins, such as *SNCA*, *PRKN*, *PINK1*, *UCHL1*, *LRRK2*, *ATP13A2*, *GBA*, *VPS35*, *DJ-1/PARK7*, *PLA2G6*, *SYNJ1*, *DNAJC6*, and *FBXO7*, associated with a familial form of Parkinson’s disease has been an important input in the basic research of this disease by revealing the association of specified proteins to the disease [49,50,51]. For example, mutations of the alpha-synuclein gene generate the formation of neurotoxic oligomers that induce oxidative stress, synaptic dysfunction, autophagy impairment, mitochondrial dysfunction, endoplasmic reticulum stress [52]. However, these mutations do not explain the role of these proteins in idiopathic Parkinson’s disease where the patients do not have these mutations.

The question is why neuromelanin-containing dopaminergic neurons degenerate and what initiates the process. The possible endogenous neurotoxins triggering the degenerative process in Parkinson’s disease include neurotoxic alpha-synuclein oligomers, DOPAL, and aminochrome generated during the oxidation of dopamine to neuromelanin [53].

### 3.1. Alpha-Synuclein Oligomers

Alpha-synuclein aggregates in two different ways: (i) to form alpha-synuclein fibrils that are not considered neurotoxic but seem to be involved in prion-like propagation of these aggregates [35]. Alpha-synuclein fibrils are one of the major components of Lewy bodies, which is a hallmark of Parkinson’s disease. Lewy bodies are also composed of a large number of proteins from mitochondria, autophagy and proteasome systems, and gene products associated to familial forms of Parkinson disease such as PINK-1, LRRK2, DJ1, and [54,55,56]. The role of Lewy bodies in Parkinson’s disease pathogenesis is controversial since it has been proposed to play key role in the propagation of the disease from one to another region [35]; on the other side, the formation of Lewy bodies is not required to induce two familial forms of Parkinson’s disease, because patients with LRRK2 and parkin mutation do not develop Lewy bodies [57,58]. Lewy bodies have been found to be present in postmortem material of Parkinson’s disease patients, where the melanin-containing dopaminergic neurons involved in the motor symptoms were lost long before. When a neuron dies the microglia phagocyte and remove all cell components of the degenerated neuron, and therefore, the postmortem material includes the tissue that survives the degenerative process [59]. It has been proposed that Lewy bodies play a neuroprotective role in Parkinson’s disease [54] by preventing the formation of neurotoxic alpha-synuclein oligomers. (ii) Alpha-synuclein is found membrane bound or in soluble state that can also aggregate to pre-fibrillar transitional species called oligomers. Alpha-synuclein structure modification plays an important role in its aggregation to oligomers. Point mutations in human alpha-synuclein DNA sequence change alpha-synuclein folding, inducing the formation of neurotoxic oligomer associated a familial form of Parkinson’s disease. However, these points mutations cannot explain alpha-synuclein aggregation to neurotoxic oligomers in idiopathic Parkinson’s disease. Another way to disrupt normal alpha-synuclein folding is the formation of adducts with some molecules, such as aminochrome that induce the formation of neurotoxic oligomers [60]. However, in vitro experiments showed that the formation of alpha-synuclein oligomers is not restricted to soluble monomer aggregation because it has been reported that alpha-synuclein fibrils ends are able to release oligomers. Short fibrils release more oligomers than long fibrils and, therefore, are more toxic [61].

### 3.2. 3,4-Dihydroxyphenylacetaldehyde (DOPAL)

Excess of cytosolic dopamine in dopaminergic neurons is degraded by the enzyme monoamine oxidase by catalyzing the oxidative deamination of dopamine. The product of this reaction, DOPAL, is converted to 3,4-dihydroxyphenylacetic acid, a reaction catalyzed by the enzyme aldehyde dehydrogenase-1. A study performed with postmortem brain tissue from Parkinson’s disease patients revealed that the aldehyde dehydrogenase-1 protein level was decreased in these samples in comparison with control human brains [62]. The decrease in aldehyde dehydrogenase-1 expression will result in accumulation of DOPAL that can be oxidized to ortho-semiquinone and later to ortho-quinone species. DOPAL has been reported to be neurotoxic by promoting oxidative stress and stimulating alpha-synuclein aggregation to oligomers [63,64,65]. However, the decrease of aldehyde dehydrogenase-1 in postmortem Parkinson’s disease material is not a direct consequence of degeneration of neuromelanin-containing dopaminergic neurons in the nigrostriatal system, because the decrease in the expression of aldehyde dehydrogenase-1 expression was measured in the neurons that had survived the degenerative process. The neuromelanin-containing dopaminergic neurons lost during years of neurodegeneration of the nigrostriatal system would have been removed by microglia long before the level of expression of aldehyde dehydrogenase was measured [66,67].

### 3.3. Aminochrome: The Neuromelanin Precursor

**Figure 2:**
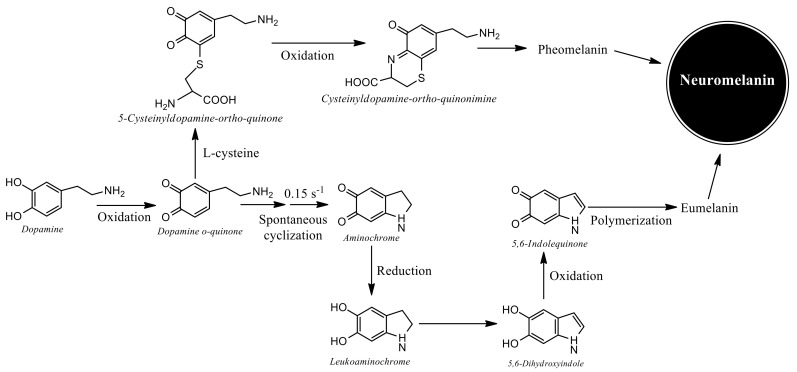
Synthesis of melanin. The synthesis of neuromelanin requires the oxidation of the catechol group of dopamine, where three ortho-quinones are generated in a sequential manner (dopamine ortho-quinone, aminochrome, and 5,6-indolequinone). Dopamine ortho-quinone is the first intermediate formed when the hydroxy groups of dopamine are oxidized. This ortho-quinone is stable at a pH lower than 2.0, which implies that at physiological pH in the cytosol it cyclizes in two steps forming aminochrome at a rate of 0.15 s^−1^. Aminochrome is the ortho-quinone most stable quinone that finally, after 40 min via oxidation, rearranges its structure generating 5,6-indolequinone at rate of 0.06 min^−1^, which is the direct precursor of neuromelanin and finally polymerizes to neuromelanin [48,68].

A possible explanation for the neurotoxic effect of aminochrome is that neuromelanin synthesis requires the formation of neurotoxic ortho-quinones [68,69]. Neuromelanin is synthesized in dopaminergic neurons when the catechol structure of dopamine is oxidized by dioxygen, metals, and enzymes, generating three ortho-quinones: dopamine ortho-quinone, which is stable only at pH lower than 2.0 and, therefore, immediately cyclizes to aminochrome. Aminochrome is a more stable molecule and is the most extensively investigated of the ortho-quinones. Finally, aminochrome can be further oxidized to 5,6-indolequinone, which can polymerize to generate neuromelanin (Figure 2).

Human neuromelanin-containing dopaminergic neurons in the nigrostriatal system play a crucial role in movement control and the loss of these neurons in substantia nigra pars compacta of more than 60% induces motor symptoms in Parkinson’s disease. Dopaminergic neurons located in substantia nigra pars compacta in the midbrain send projections to the dorsal striatum in the forebrain. Interestingly, neuromelanin accumulates with the age in the cell body of dopaminergic neurons of substantia nigra pars compacta due to these neurons have lower expression of VMAT-2 in comparison with dopaminergic neurons of mesolimbic system located in ventral tegmental area [70]. VMAT-2 mediated transport of dopamine into monoaminergic vesicles is essential to prevent dopamine oxidation to neuromelanin. Dopamine within monoaminergic vesicles is completely stable due the slight low pH environment that prevents dopamine oxidation to neuromelanin.

Human neuromelanin is composed by pheomelanin and eumelanin in a ratio of 1:3. These different melanin structures arise from the existence of two different pathways of melanin synthesis. The eumelanin synthesis involves dopamine oxidation to dopamine orthoquinone → aminochrome → 5,6-indolequinone → eumelanin, while pheomelanin synthesis encompasses dopamine oxidation to dopamine ortho-quinone, which, in the presence of L-cysteine, generates 5-cysteinyldopamine. 5-Cysteinyldopamine is oxidized to 5-cysteinyldopamine ortho-quinone. The cysteinyl group is oxidized to cysteinyldopamine ortho-quinonimine, which finally polymerizes to pheomelanin. Glutathione conjugation of dopamine ortho-quinone forms 5-glutathionyldopamine that is degraded to 5-cysteinyldopamine, which polymerizes to pheomelanin or is excreted [71]. 5-Cysteinyldopamine is a stable final product that can be detected in serum or urine of melanoma patients [72] (Figure 2).

The most neurotoxic of these ortho-quinone is aminochrome, which induces (i) oxidative stress by being reduced with a single electron to leukoaminochrome *o*-semiquinone radical [73]; (ii) the formation of neurotoxic oligomers of alpha-synuclein [60]; (iii) mitochondrial damage including membrane impairment and inhibition of complex I that ultimately leads to mitochondrial dysfunction [73,74,75,76,77]; (iv) neuroinflammation [78,79]; (v) stress of the endoplasmic reticulum [80]; and (vi) impairment of protein degradation of both lysosomal and proteasomal systems [81,82]. Aminochrome is not a stable molecule that can be secreted from dopaminergic neurons to neighboring neurons, generating a propagative neurotoxic effect, since aminochrome immediately after its formation can be reduced by flavoenzymes or form adducts with proteins such as mitochondrial complex I, alpha-synuclein, actin, alpha- and beta-tubulin in cytoskeleton, and lysosomal vacuolar-type H + -ATPase, among other proteins [60,74,83,84]. Therefore, aminochrome cannot induce a propagative neurotoxic effect, but rather induces a neurotoxic effect focused on a single dopaminergic neuron.

The synthesis of neuromelanin in dopaminergic neurons is a harmless and normal process because neuromelanin formation increases with age in the substantia nigra, and healthy older adults at death have these neurons intact loaded with this dark pigment. This observation seems paradoxical, since neuromelanin synthesis in dopaminergic neurons generates the neurotoxic aminochrome, while neuromelanin synthesis generally is a harmless process. The explanation for this apparent contradiction is that there are neuroprotective mechanisms that prevent the neurotoxic effects of aminochrome in healthy individuals, who have their neuromelanin-containing dopaminergic neurons intact in the nigrostriatal system. There are two neuroprotective enzymes that prevent aminochrome-dependent neurotoxicity, DT-diaphorase and GST M2-2.

### 3.4. DT-Diaphorase

DT-diaphorase (NQO1; NAD(P)H: quinone oxidoreductase; EC.1.6.99.2) is the unique flavoenzyme that reduces quinones to hydroquinones by transfer of two electrons from NADH or NADPH [85]. Other flavoenzymes catalyze one-electron reduction of quinones to generate semiquinone radicals that in general are very reactive with oxygen. DT-diaphorase catalyzes the two-electron reduction of aminochrome to leukoaminochrome by using NADH or NADPH as electron donors. Leukoaminochrome can slowly autoxidize in the presence of dioxygen generating superoxide, but the presence of superoxide dismutase inhibits the auto-oxidation of leukoaminochrome, and glutathione peroxidase removes H_2_O_2_ formed in this autoxidation. DT-diaphorase is expressed in different organs, and in the brain, the enzyme is expressed in substantia nigra, striatum, hypothalamus, hippocampus, frontal cortex, ventral tegmental area, and cerebellum. Interestingly, DT-diaphorase is responsible for 97% of the total quinone reductase activity in the substantia nigra [86].

DT-diaphorase occurs in both dopaminergic neurons and astrocytes, but glutathione transferase M2-2 is expressed only in astrocytes. The oxidative pathway of dopamine oxidation to neuromelanin (dopamine → dopamine ortho-quinone → aminochrome → 5,6-indolequinone → neuromelanin) exists in dopaminergic neurons of substantia nigra where the expression of VMAT-2 is not enough high to complete prevent dopamine oxidation in the cytosol. Dopamine inside monoaminergic vesicles is completely unreactive because the protons of hydroxyl groups are firmly bound. Dopamine transport into monoaminergic vesicles mediated by VMAT-2 is coupled to an ATPase that pumps protons, decreasing the pH of the vesicles. The level of VMAT-2 expression in dopaminergic neurons of the mesolimbic system is much higher than in nigral neurons, preventing neuromelanin formation. Astrocytes express both dopamine transporter and nonspecific transporter that take up dopamine released under neurotransmission, which can be oxidized to dopamine ortho-quinone and aminochrome. However, glutathione transferase M2-2 catalyzes glutathione conjugation of both dopamine ortho-quinone and aminochrome, preventing the formation of 5,6-indolequinone, which is the direct precursor of neuromelanin. Astrocytes do not have dopamine synthesis and the level of dopamine in astrocytes cytosol depends on the competition between dopamine re-uptake mediated by dopamine transporter into dopaminergic neurons and dopamine uptake into astrocytes of dopamine released during neurotransmission [85].

**Figure 3:**
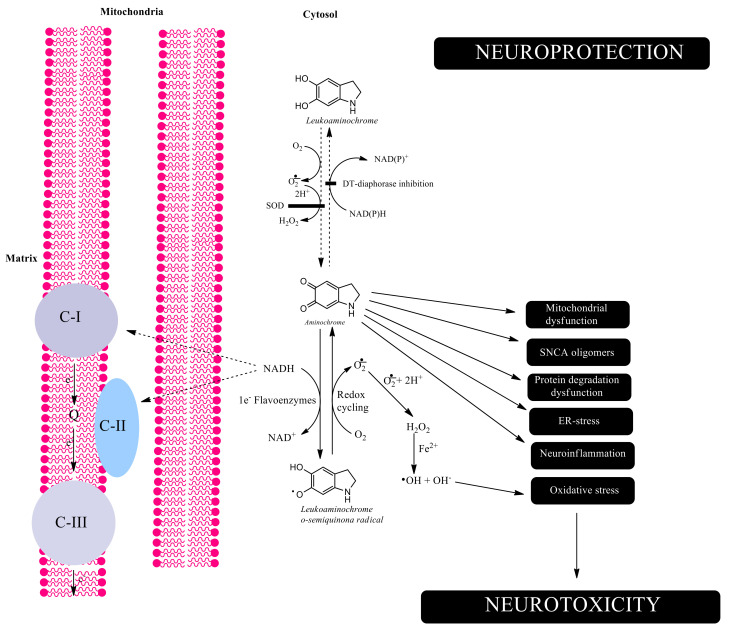
Aminochrome-induced neurotoxicity. DT-diaphorase catalyzes the reduction of aminochrome with two electrons to leukoaminochrome, preventing the neurotoxic effects of aminochrome. Leukoaminochrome can autoxidize very slowly in the presence of dioxygen, generating superoxide. However, the autoxidation of leukoaminochrome is significantly faster in the presence of superoxide and the presence of superoxide dismutase in the cytosol inhibits the autoxidation, since it removes the superoxide molecules, which are the true motor of the autoxidation of leukoaminochrome. Inhibition of DT-diaphorase allows aminochrome to be neurotoxic in two different ways: (i) aminochrome can be reduced with one-electron donors to leukoaminochrome *o*-semiquinone, which is extremely reactive with dioxygen generating superoxide. Reduction of aminochrome with a single electron generates a redox cyclization between aminochrome and leukoaminochrome o-semiquinone radical, which depletes dioxygen and NADH. NADH depletion affects the activity of the mitochondrial respiratory chain and ultimately ATP production. Superoxide spontaneously or in the presence of superoxide dismutase is converted to hydrogen peroxide, which in the presence of reduced iron (Fe^2+^) generates hydroxyl radicals. The hydroxyl radical is a powerful reactive oxygen species that induces oxidative stress; and (ii) aminochrome forms adducts with different proteins such as alpha-synuclein, complex-I in the mitochondrial respiratory chain, and other cell components. Aminochrome induces mitochondrial dysfunction, formation of neurotoxic alpha-synuclein oligomers, dysfunction of both lysosomal and proteasomal protein degradation systems, endoplasmic reticulum stress and neuroinflammation.

DT-diaphorase competes with other flavoenzymes that reduce aminochrome in a one-electron process to leukoaminochrome *o*-semiquinone radical, which is extremely reactive with oxygen, as evidenced by experiments performed with electron spin resonance [87]. One-electron reduction of aminochrome induces the generation of redox cycling between aminochrome and leukoaminochrome *o*-semiquinone radical by reducing dioxygen to superoxide until dioxygen and NADH are depleted. This redox cycle is a potent inducer of oxidative stress and depletion of NADH that inhibits mitochondrial electron transport and ATP formation. The silencing of DT-diaphorase expression by using a siRNA targeting this gene induces aminochrome neurotoxicity, supporting the notion of a protective role of this enzyme [88]. Thus, two-electron reduction of aminochrome catalyzed by DT-diaphorase prevents oxidative stress and mitochondrial electron transport chain inhibition caused by NADH depletion [73]. Another neurotoxic mechanism caused by aminochrome involves its ability to induce the formation of alpha-synuclein neurotoxic oligomers, which have been proposed to induce mitochondrial dysfunction, autophagy dysregulation, oxidative stress, and endoplasmic reticulum stress [52,60]. DT-diaphorase prevents the formation of neurotoxic alpha-synuclein oligomers [60]. DT-diaphorase also thwarts aminochrome-induced proteasomal dysfunction [89] as well as aminochrome-induced lysosomal dysfunction that it is essential to perform autophagy-dependent protein degradation [83]. Aminochrome-induced aggregation of alpha- and beta-tubulin prevents microtubules assembly and stability. DT-diaphorase also prevents aminochrome-induced cytoskeleton disruption by inhibiting actin and alpha- and beta-tubulin aggregation, which dramatically affects neurons morphology due to a phenomenon called cell shrinkage [84]. Microtubules play an important role in neuron cytoskeleton structure, as well as an essential role in axonal transport of proteins and neurotransmitter vesicles that is strongly decreased in animals treated with aminochrome [90] (Figure 3). Furthermore, microtubules are involved in the fusion between lysosomes and autophagosomes that it is crucial for autophagy-dependent protein degradation, and DT-diaphorase prevents tubulin aggregation caused by aminochrome [82].

### 3.5. Glutathione Transferase M2-2

Glutathione is an important antioxidant in neurons, but also plays an important protective role in Parkinson’s disease by participating in aminochrome conjugation catalyzed by glutathione transferase [85]. Human class Mu glutathione transferases catalyze aminochrome glutathione conjugation where GST M2-2 is 193- and 1000-times more active than GST M1-1 and GST M3-3 enzymes, respectively. Human glutathione transferase M2-2 is expressed in astrocytes but not in neurons and aminochrome induces an increase in the expression of this enzyme [26]. GST M2-2 catalyzes aminochrome conjugation to 4-S-glutathionyl-5,6-dihydroxyindoline that is resistant to biological oxidants such as dioxygen, superoxide and hydrogen peroxide, suggesting that this is a final product that it is not able to contribute to neurotoxic oxidation and reduction reactions [32].

**Figure 4:**
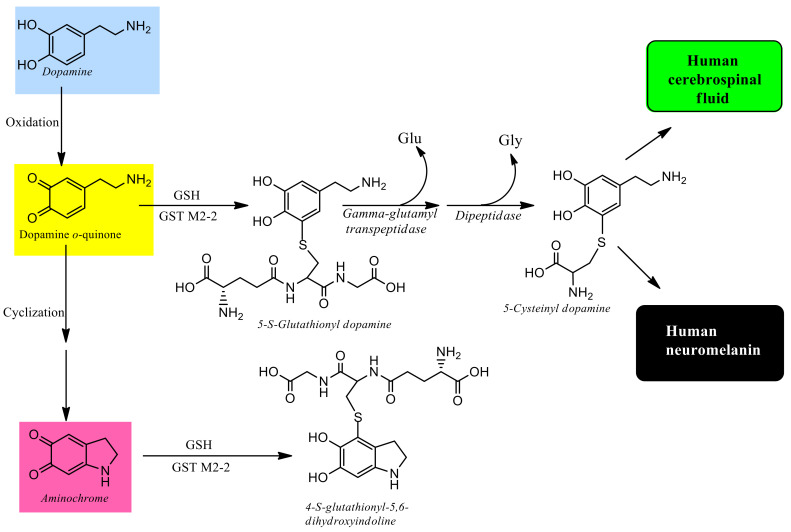
Aminochrome conjugation with glutathione. Glutathione transferase M2-2 catalyzes glutathione conjugation of dopamine *o*-quinone to 5-S-glutathionyl dopamine, which degrades to 5-cysteinyl dopamine. 5-cysteinyl dopamine has been found in neuromelanin and human spinal brain fluid, suggesting that it is a final product. Glutathione transferase M2-2 also catalyzes the conjugation of aminochrome with glutathione to 4-S-glutathionyl-5,6-dihydroxyindoline, which is resistant to biological oxidizing agents such as dioxygen, superoxide, and hydrogen peroxide. The aminochrome conjugation prevents neurotoxic effects and appears to be a major neuroprotective mechanism.

GST M2-2 also catalyzes glutathione conjugation of dopamine *o*-quinone to 5-glutathionyl dopamine [91]. In general, all glutathione conjugates are degraded by the enzyme γ-glutamyl transpeptidase and dipeptidase. 5-Glutathionyldopamine is degraded to 5-cysteinyldopamine which has been found in human neuromelanin of substantia nigra, cerebrospinal fluid and other dopamine-rich regions, such as putamen, globus pallidus and caudate nucleus [92,93,94]. These data suggest that 5-cysteinyldopamine is a final product, supporting the notion that GST M2-2 is a neuroprotective enzyme against aminochrome neurotoxicity [81] (Figure 4).

## 4. Astrocytes Protects Dopaminergic Neurons

There is an interrelation between neurons and astrocytes, where astrocytes secrete glutathione and cysteine that neurons take up to maintain stable levels of thiols to prevent oxidative stress generated by the loss of electrons from the mitochondrial transport chain. However, this transfer of glutathione and cysteine is only in one direction, from astrocytes to neurons [95]. The interrelation between neurons and astrocytes is not restricted to exchange of glutathione and cysteine. Astrocytes play an important role in neuronal survival and function by supporting neuron energy demand by providing lactate, a precursor of glucose synthesis in the gluconeogenesis pathway, by generating astrocyte-neuron lactate shuttle [96]. Neurons are completely dependent on ATP availability for essential functions, such as neurotransmission or axonal transport, and glucose is the main source of energy production under normal conditions in the brain. Astrocytes also play an important role by taking up lipid droplets generated by neurons. Astrocytes detoxify neurotoxic fatty acids created in hyperactive neurons that transport these fatty acids into astrocytes lipid droplets by using apolipoprotein E-positive lipid particles. Astrocytes’ mitochondrial β-oxidation oxidizes these neuronal fatty acids and induces detoxifying enzyme expression [97]. Another interrelation between astrocytes and neurons is glutamate-glutamine metabolism. An important part of release glutamate by glutaminergic neurons is take up by astrocytes due to neurons have lower capacity to take up glutamate. Astrocytes convert most of the glutamate to glutamine that neurons take up [98].

**Figure 5:**
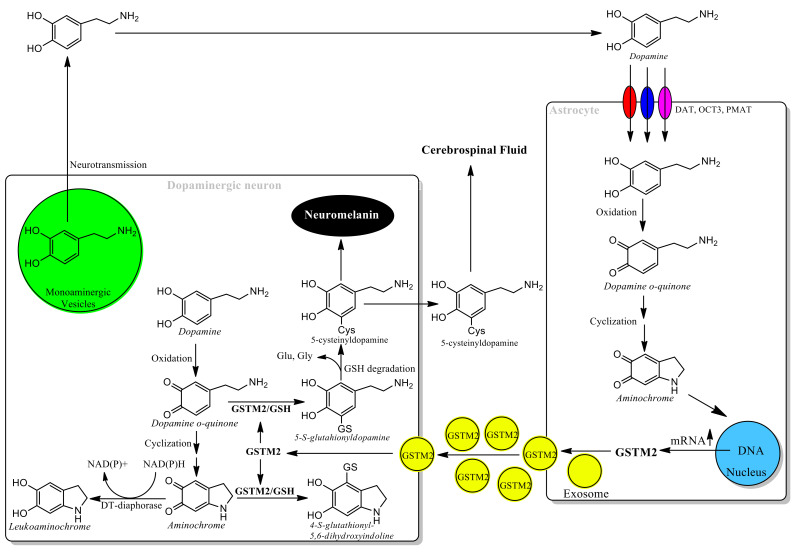
Astrocytes protects dopaminergic neurons against aminochrome neurotoxicity. A new mechanism of neuroprotection of dopaminergic neurons against the neurotoxic effects of aminochrome mediated by astrocytes has been reported. Dopaminergic neurons express DT-diaphorase to prevent aminochrome neurotoxic effects. Dopamine neurons release dopamine normally during the neurotransmission process, which astrocytes are also able to take up through transporters that are expressed in astrocytes such as dopamine transporter (DAT), organic cation transporter-3 (OCT3), and plasma membrane monoamine transporter (PMAT). Dopamine within astrocytes can be oxidized to form aminochrome, which increases the expression of glutathione transferase M2-2 (GSTM2). GST M2-2 prevents the neurotoxic effects of aminochrome within astrocytes by conjugating it with glutathione, but GST M2-2 is also exported through exosomes that are released from astrocytes into dopaminergic neurons. Exosomes loaded with GST M2-2 penetrate dopaminergic neurons, releasing this enzyme into the cytosol of these neurons, where together with DT-diaphorase they prevent the neurotoxic effects of aminochrome.

A new interrelation between neurons and astrocytes, in order to protect dopaminergic neurons against aminochrome neurotoxicity, has been reported [25,26,85,99,100]. DT-diaphorase and GST M2-2 play key neuroprotective roles against aminochrome neurotoxicity in neuromelanin-containing dopaminergic neurons in the nigrostriatal system, explaining why neuromelanin synthesis is a normal and harmless pathway. DT-diaphorase is expressed in dopaminergic neurons and astrocytes, while GST M2-2 is expressed only in human astrocytes. Astrocytes surrounding dopaminergic neurons can take up dopamine released during neurotransmission through the dopamine transporter and other non-specific transporters such as organic cation transporter-3 and plasma membrane transporter. Dopamine within astrocytes can be oxidized, thereby generating aminochrome that induces an increase in the expression of GST M2-2. This enzyme conjugates aminochrome with glutathione within astrocytes, but, in addition, astrocytes secrete GST M2-2 through exosomes [85]. GST M2-2 loaded exosomes penetrate dopaminergic neurons to prevent aminochrome neurotoxicity in collaboration with DT-diaphorase (Figure 5). Significantly, a study of senescence processes demonstrated that small extracellular vesicles from young human donors contained GST M2-2 which could ameliorate aging of old fibroblasts seemingly mimicking the protective effect of astrocytes on neurons.

## 5. Conclusions

The protective mechanism against aminochrome neurotoxicity, where astrocytes collaborate with dopaminergic neurons, plays a crucial role in preventing neurotoxic effects during neuromelanin synthesis where aminochrome is generated. This protective system composed, of DT-diaphorase and GST M2-2, explains why neuromelanin synthesis is a harmless and normal chemical pathway, and why neuromelanin-containing dopaminergic neurons of the nigrostriatal system are intact in healthy seniors.
